# Phenotypic and genetic changes of Crimean-Congo hemorrhagic fever virus during serial passage in susceptible cell lines

**DOI:** 10.1007/s00705-025-06420-4

**Published:** 2025-11-07

**Authors:** Nóra Deézsi-Magyar, Bereniké Novák, Gyula Zsidei, Norbert Solymosi, Marianna Mezősi-Csaplár, Dániel Déri, Bernadett Pályi, Zoltan Kis

**Affiliations:** 1 National Biosafety Laboratory, National Center for Public Health and Pharmacy, Budapest, Hungary; 2 School of PhD Studies, Semmelweis University, Budapest, Hungary; 3 University of Veterinary Medicine Budapest, Budapest, Hungary; 4 Institute of Medical Microbiology, Faculty of Medicine, Semmelweis University, Budapest, Hungary

**Keywords:** Crimean-Congo hemorrhagic fever virus, Adaptation, *In vitro *testing, Serial passaging, Whole-genome sequencing, Genetic changesGrowth-induced mutations

## Abstract

Background Single-stranded RNA viruses such as the Crimean-Congo hemorrhagic fever virus (CCHFV) exhibit a high degree of genetic diversity that can result in the emergence of new viral quasispecies. The aim of this study was to examine the kinetics of CCHFV replication and to identify genetic variations resulting from serial passage in susceptible cell lines that could potentially affect the infectivity and fitness of laboratory virus strains. As efficacy studies of candidate therapeutics and vaccines rely on well-characterized virus stocks, mutations emerging due to virus propagation might result in inaccurate test results. Methods Viral growth kinetics were examined in four cell lines (Vero E6, Vero, SW13, and BHK-21), using different multiplicity-of-infection values. After 10 rounds of serial passaging and cross-passaging, whole-genome sequencing and bioinformatic analysis were performed to map growth-adaptive signature mutations. Results All four cell lines were found to be susceptible to CCHFV infection, and permissivity increased during serial passaging in Vero and BHK-21 cells. Mutations emerged in a cell-line-specific manner, and the particular cell line used for virus propagation had a significant effect on the mutation frequency, especially in the L segment. Conclusions By mapping mutations that occurred during serial passage, we were able to observe viral evolution in a controlled laboratory setting, as well as the accompanying phenotypic changes. Our results provide information about how CCHFV adapts to commonly used cell lines that might be applied for the development of diagnostic tests, antiviral drugs, and vaccines.

**Supplementary Information:**

The online version contains supplementary material available at 10.1007/s00705-025-06420-4.

## Introduction

Crimean-Congo hemorrhagic fever (CCHF) is a widespread tick-borne disease that can cause various symptoms, including hemorrhagic manifestations, in humans [1]. The reported fatality rate among hospitalized patients is 9–40%, and the number of annual cases and seroprevalence is increasing worldwide [1, 2]. The causative agent, Crimean-Congo hemorrhagic fever virus (CCHFV; species *Orthonairovirus haemorrhagiae*, genus *Orthonairovirus*, family *Nairoviridae*), is endemic to the Balkan region, Eastern and Southern Europe, the Middle East, Africa, and Asia [3, 4]. Currently, there are no licensed therapeutics or vaccines available for CCHF [1, 5]. Consequently, the World Health Organization (WHO) has identified CCHFV as a priority pathogen that poses a great public-health risk due to its epidemic potential and/or the lack of sufficient countermeasures [5, 6]. The WHO Research and Development Blueprint promotes the development of diagnostics, therapeutics, vaccination, and vector control strategies against CCHFV that require standardized, well-characterized, and reproducible laboratory models and protocols at the level of applied and fundamental research [5, 6].

The aim of this study was to identify mutations that occur during adaptation of CCHFV to cell lines that are likely to be used for virus isolation and production of virus stocks for genome-based research and to assess their affect on viral infectivity. This is a particularly important quality management task in the case of single-stranded RNA viruses, which have high mutation rates due to the lack of proofreading activity in their RNA-dependent RNA polymerase (RdRp) [7, 8]. Furthermore, genetic variations may lead to changes in the infectivity and viral fitness of strains that are used in laboratory experiments [7]. Because efficacy studies of potential therapeutic agents and vaccines rely on laboratory virus stocks, mutations emerging during virus propagation can potentially result in inaccurate test results [8].

The CCHFV genome consists of three RNA segments of negative polarity with complementary non-coding regions present at the 5’ and 3’ termini of the segments [9, 10]. The small segment (S; 1.6 kb) encodes the nucleocapsid protein (NP) and the non-structural S protein (NS_S_). Because NS_S_ is encoded in the opposite orientation relative to the NP gene, CCHFV may be considered an ambisense virus [10]. The medium segment (M; 5.4 kb) encodes the glycoprotein complex (GPC) in a single open reading frame. The GPC is processed post-translationally into several structural and non-structural proteins, including Gn and Gc, which are present on the surface of the virion and are responsible for receptor binding and virus entry. The NP, together with the viral RNA and the RdRp (or L protein), which is encoded by the large segment (L; 12.1 kb), form the ribonucleoprotein complex inside the host-derived membrane envelope [9, 10].

CCHFV is a genetically diverse virus with a wide geographical distribution. Although the NP and L proteins are conserved among all CCHFV strains, with approximately 95% amino acid sequence identity, the GPC is much more variable, with less than 75% amino acid sequence conservation [11]. Although there are multiple genetic clades that correspond to different geographical locations, the nature of the selective pressures driving the genetic diversity of the virus is still unknown [10, 11]. Phylogenetically, CCHFV is divided into seven genetic clades: two from Asia, two from Europe, and three from Africa [11]. The mutation rates are estimated to be 1.09 × 10^−4^, 1.52 × 10^−4^, and 0.58 × 10^−4^ substitutions/site/year for the S, M, and L segment, respectively [12].

Inter- and intrahost genetic diversity of RNA viruses can be described by the complex interactions between *de novo* mutations and selection, which includes adaptation to the host organism, including evasion of host immunity [7]. A frequently used method for studying virus diversity is to propagate the virus over many generations under conditions resulting in severe genetic bottlenecks in order to reduce the effectiveness of selection. As a result, mutations within the viral genome can accumulate in an unbiased manner at the basic mutation rate of the virus [7]. One very commonly employed method for producing a genetic bottleneck is to infect susceptible cells at a very low multiplicity of infection (MOI; the ratio of infectious virus particles to cells), which favors the selection of the fittest genomes within the viral population [7].

Although many different cell lines are used for *in vitro* propagation of CCHFV [13, 14], the viral mutation rate and the variation frequency (VF) in many of these cell lines are still unknown. This study focuses on the most frequently used cell lines in CCHFV research and the effect of *in vitro* serial passaging on the infectivity and genetic composition of the virus.

## Materials and methods

### Cell cultures

Four different cell lines that are susceptible to CCHFV infection were included in this study. Vero E6 (European Collection of Cell Cultures [ECACC], Porton Down, Salisbury, Wiltshire, United Kingdom), Vero (Nuvonis Technologies GmbH, Vienna, Austria), and SW13 (ECACC) cells were maintained in Dulbecco's modified Eagle medium (DMEM; VWR International bv, Leuven, Belgium) supplemented with 10% fetal bovine serum (FBS; Euroclone S.p.A., Pero, Italy) at 37 °C with 5% CO_2_. BHK-21 cells (ECACC) were grown in Glasgow minimum essential medium (GMEM; Biowest SAS, Rue de la Caille, France) supplemented with 10% FBS at 37 °C with 5% CO_2_. In order to minimize bias introduced by cell passaging and morphological changes, cells were passaged at least two times and no more than 15 times, and reagents and culture flasks with the same lot number were used during the course of the experiment.

### Virus growth kinetics and passage in cell culture

The CCHFV strain Afg09-2990, provided by the Bernhard Nocht Institute for Tropical Medicine (Hamburg, Germany) through the European Virus Archive (EVA), was used in this study [8]. The inoculum virus stock (passage 0; P0) was prepared from an infected Vero E6 cell supernatant at the National Biosafety Laboratory, National Center for Public Health and Pharmacy (Budapest, Hungary), under biosafety level 4 (BSL-4) conditions. The viral RNA copy number and 50%tissue culture infectious dose (TCID_50_) per mL of the initial virus stock were determined using quantitative reverse transcription polymerase chain reaction (RT-qPCR) and virus titration, respectively, as described below. The virus was propagated in 6-well plates (TPP Techno Plastic Products AG, Trasadingen, Switzerland). For the first passage (P1), a suspension of each cell Line at an initial density of 3 × 10^5^ cells/well was inoculated in three biological and three technical replicates, using three different multiplicity of infection values (MOI 0.005, 0.01, and 0.1). Supernatant samples were collected immediately after inoculation (day 0 postinfection [dpi]) and then at 24-hour intervals until day 7. Prior to titration, the samples were stored at −80°C. The viral RNA copy number and TCID_50_ per mL of each sample were calculated as described below.

Based on virus growth, the MOI that resulted in the highest logarithmic titer increase and the time point with the highest TCID_50_ per mL were determined. After the first passage, the virus was further propagated in the same cell line for an additional four passages (P2-P5) using the previously determined optimal MOI and time point for harvesting the supernatant (Supplementary Fig. S1). Thereafter, the virus was cross-passaged in each of the cell lines for an additional five rounds (P6-P10). During the first cross-passage (P6), the viral growth kinetics were assessed again, and the optimal MOI and time point for harvesting the virus were re-evaluated, and subsequent passages were carried out accordingly (P7-P10). The viral RNA copy number and infectious titer per mL were determined for each sample after every passage. During the final passage (P10), the viral growth kinetics were again examined as described above.

### Nucleic acid extraction and RT-qPCR

Nucleic acid was extracted from a 50-µL cell culture supernatant by adding 280 µL of AVL buffer (QIAGEN, Hilden, Germany). After 10 minutes of incubation at room temperature, 280 µL of ethanol (Szkarabeusz Laboratóriumi Kft., Pécs, Hungary) was added to each sample. Viral RNA was extracted using a Chemagic Viral DNA/RNA Kit special H96 (PerkinElmer, Waltham, Massachusetts, USA) on a Chemagic 360 instrument (PerkinElmer, Waltham, Massachusetts, USA) using the Chemagic Viral 300 360 H96 prefilling 18 min VD210204.che protocol. RT-qPCR was conducted on a LightCycler 480 Instrument II platform (Hoffmann-La Roche, Basel, Switzerland), using a LightCycler Multiplex RNA Virus Master kit (Hoffmann-La Roche, Basel, Switzerland). The applied primers and probes specific for the S segment (clade IV) were described previously by Sas et al. [15]. To quantify the M and L segments, primers and probes specific for the respective segments (Supplementary Table S1) were designed in-house using the Geneious Prime 2021.2.2 software (Biomatters, Auckland, New Zealand).

### Virus titration

SW13 cells in DMEM with 10% FBS were seeded into 96-well plates (TPP Techno Plastic Products AG, Trasadingen, Switzerland) at a density of 10 × 10^4^ cells/well. The cells were then inoculated in triplicate with tenfold serial dilutions of each sample. After incubation for 5 days at 37 °C with 5% CO_2_, the supernatants were collected, and the cell monolayers were inactivated and fixed with 4% formaldehyde solution. Fixed cells were stained using crystal violet to observe the cytopathic effect (CPE). Infectious titers were calculated by the Spearman and Kärber method [16].

### Whole-genome sequencing

Viral RNA extracted from the cell culture supernatants with the highest identified TCID_50_ per mL of P0, P5, P6, and P10 samples was subjected to DNase I digestion (Turbo DNase; Invitrogen, Thermo Fisher Scientific, Waltham, Massachusetts, USA), purified using AMPureXP beads (Beckman Coulter, Brea, California, USA) according to manufacturer’s instructions, and eluted in 10 µL of nuclease-free water. To avoid primer bias, randomly amplified DNA products were prepared from the purified RNA samples of all three biological replicates using a single-primer sequence-independent amplification (SISPA) protocol and K/K8N primers as described previously [17]. After enrichment, parallel replicates were pooled together for Illumina Nextera XT V2 library preparation (Illumina, Waltham, Massachusetts, USA) and sequenced on an Illumina MiSeq instrument (Illumina, Waltham, Massachusetts, USA), using 2 × 150 bp paired-end chemistry (Reagent Kit v2 Micro; Illumina, Waltham, Massachusetts, USA).

### Data analysis

Virus growth kinetics during the first passage (P1), the first cross-passage (P6), and the final passage (P10) were measured by determining by the mean virus RNA copy number per mL and geometric mean TCID_50_ per mL of the replicates at each sampling time point (0–7 dpi). Kinetic curves (x, days post-inoculation; y, virus titer) and standard deviations (SD) were visualized by using GraphPad Prism 9.5.0 software (GraphPad Software Inc., Boston, MA, USA). The particle (virus RNA copy number)-to-TCID_50_ per mL ratio was also determined during P1 at all three MOIs and during P10 (MOI 0.005) on each day postinfection.

To analyze the effect of the individual cell line, the MOI, and the passage number on virus growth during P1, P6, and P10, an ordinary two-way analysis of variance (ANOVA) was performed using GraphPad Prism 9.5.0 software. The reported *P*-values are significant at the 5% level. Based on virus growth kinetics, we determined cell line permissivity for CCHFV by comparing TCID_50_ per mL values on the day postinfection with the highest viral load, determined during P10 and P1.

For genome analysis, forward and reverse sequencing reads in fastQ files were quality trimmed using TrimGalore and mapped to reference sequences of the S (accession no. HM452305.1), M (accession no. HM452306.1), and L (accession no. HM452307.1) segments using bowtie2 and SAMtools. High-frequency sequence variations occurring in at least 10% of all reads in the regions that met the minimum sequencing depth criterion of 100x were included in the analysis. The frequency of each sequence variation was determined based on the sequencing data of parallel biological replicates pooled together prior to Library preparation. The distribution of nucleotide sequence variations by segment and genome position for each sample was determined by using R and Geneious Prime 2021.2.2 software. The average variation frequency (VF) and the number of single-nucleotide polymorphisms (SNPs) were determined for each sample and visualized using GraphPad Prism 9.5.0 software. Analysis of the nucleotide sequence variations and manual annotation of high-frequency mutations occurring with a frequency above 40% (consensus level) were performed and compared to the whole-genome sequence data for the P0 inoculum virus stock.

## Results

### MOI dependence of viral growth kinetics

**Fig. 1:**
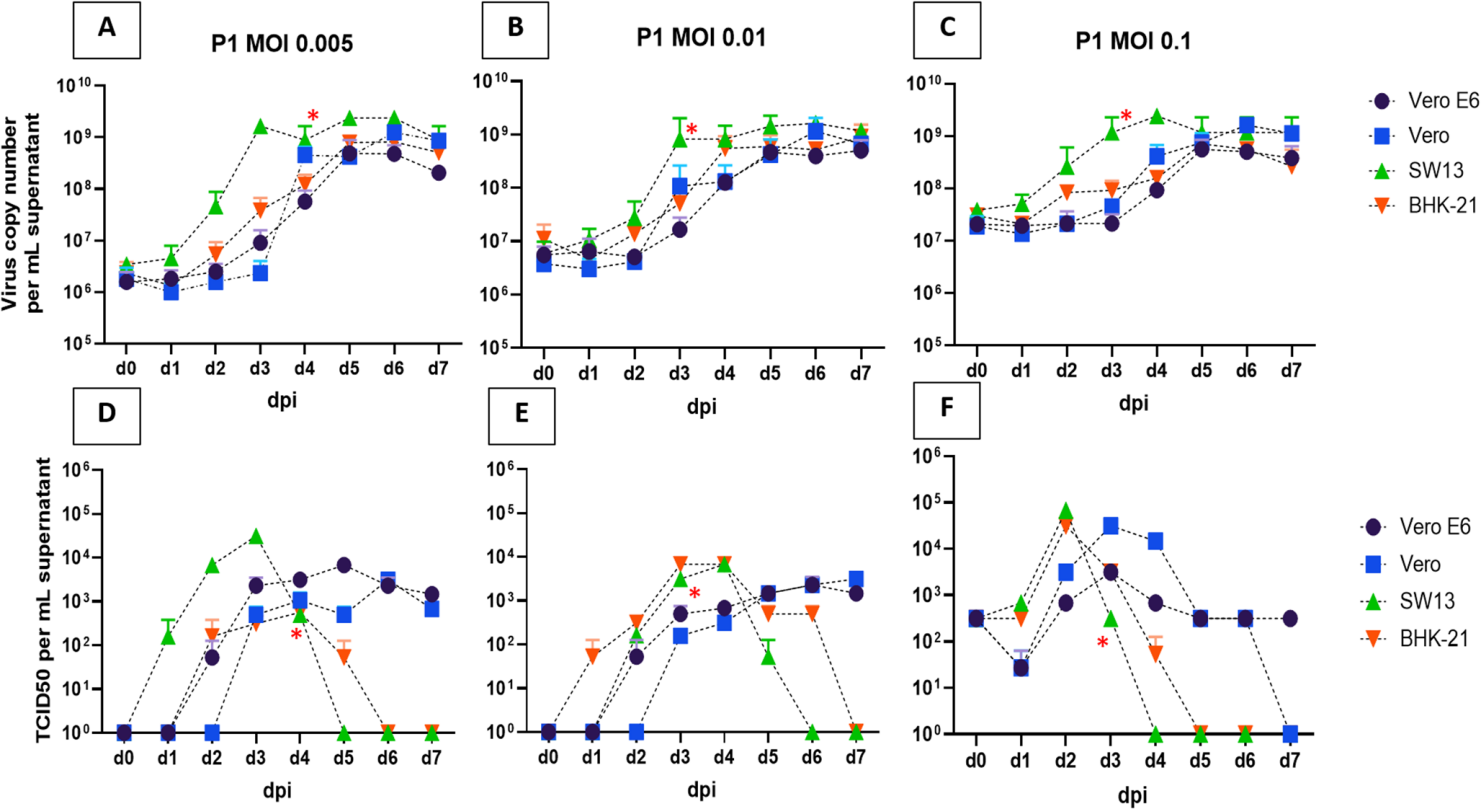
Viral growth kinetics during P1 at three different MOIs. (**A**-**C**) Kinetic curves of the viral RNA copy number per mL at an MOI of 0.005 (panel A), 0.01 (panel B), and 0.1 (panel C). (**D**-**E**) Kinetic curves of the TCID_50_ per mL of supernatant at an MOI of 0.005 (panel **D**), 0.01 (panel **E**), and 0.1 (panel **F**). The four cell lines are indicated by different colors: Vero E6 (dark blue), Vero (blue), SW13 (green), BHK-21 (orange). dpi, days postinfection. The standard deviation (SD)/geometric SD is indicated by error bars. The time point at which a full cytopathic effect (CPE) was observed in SW13 cells is indicated by a red asterisk.

During the first passage, we examined the MOI dependence of the growth kinetics of CCHFV in four different cell lines (Fig. 1). CPE caused by virus replication was detected only in SW13 cells. When comparing the cell lines, the highest virus RNA copy number per mL was observed in SW13 cells at all three MOIs (Supplementary Table S2). The virus growth curve in BHK-21 cells was similar to that in SW13 cells (Fig. 1A-C), but no CPE was observed in BHK-21 cells. Similar kinetics were seen in Vero cells and Vero E6 cells, but with, on average, a 0.457-log higher virus copy number per mL in Vero cells at all MOIs (Fig. 1A-C, Supplementary Table S2). The maximum viral RNA copy number per mL increased to the same extent at all MOIs but depended on the cell line used (Fig. 1A-C). Consequently, the logarithmic increase in virus RNA copy number per mL was higher at an MOI of 0.005 than at higher MOIs. Two-way ANOVA showed that both the MOI and the cell line had a significant effect on the logarithmic increase in virus RNA copy number (*P* < 0.0001 and *P* = 0.0319, respectively).

When comparing the infectious titers (Supplementary Table S2), the highest TCID_50_ per mL was measured when the virus was passaged in SW13 cells (Fig. 1D-F), and the titer started to decline rapidly after CPE was observed. In all of the cell Lines, the logarithmic increase in the virus titer was the highest at an MOI of 0.005. However, the maximum was reached on a later day postinfection compared to MOI 0.1 (48 h later on average, data shown in Supplementary Table S2). Interestingly, the cell line used had no significant effect on the logarithmic increase in the infectious titer (*P* = 0.2818), while the MOI affected it significantly (*P* < 0.0001).

We also examined how the time point at which the viral titer reached a maximum depended on the MOI (Supplementary Table S2). Both the MOI and the cell line showed a significant effect (*P* = 0.0039 and *P* = 0.0122, respectively). Based on the results of the MOI dependence of the virus growth kinetics, we chose an MOI of 0.005 for the subsequent (P2-P10) passages for all cell Lines and days 5 (Vero E6), 6 (Vero), 3 (SW13), and 4 (BHK-21) postinfection for harvesting the supernatant of the subsequent four passages (Supplementary Table S1).

### Effect of serial passaging and the cell line on virus growth and permissivity

During the initial cross-passage (P6), the virus growth kinetics were examined again in each cell line to determine the optimal MOI and the best time point for subsequent passages (P7-P10; Supplementary Table S3). No clear trend emerged in the maximum virus RNA copy number per mL when comparing P1, P6, and P10. However, the differences between the cell lines decreased with later passages (Supplementary Table S3). The highest values were observed when the virus was cross-passaged in SW13 cells during both P6 and P10. ANOVA analysis revealed that the cell line factor affected the logarithmic increase in viral RNA copy number per mL significantly (*P* < 0.0038) when comparing passages P1, P6, and P10 on the same cell lines (without cross-passaging).

**Fig. 2:**
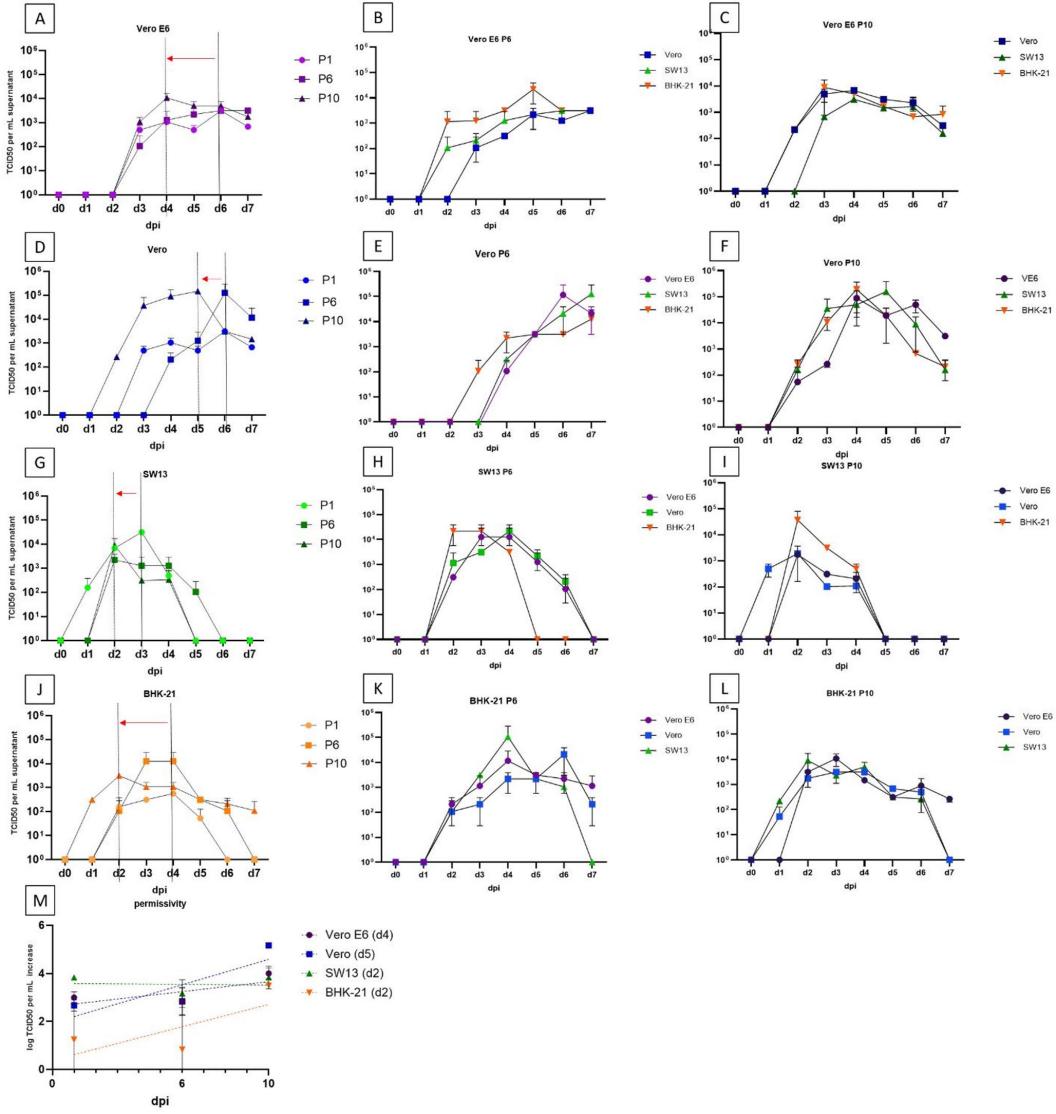
Virus growth kinetics (TCID_50_ per mL) and permissivity of different cell lines for CCHFV. The graphs are organized by new cell line, with the original cell line indicated by color (Vero E6, dark blue; Vero, blue; SW13, green; BHK-21, orange). P1, passage 1; P6, passage 6; P10, passage 10. Panels A, D, G, and J display virus growth kinetics in cell Lines without cross-propagation. The day corresponding to the highest infectious titer at an MOI of 0.005 during P1 (solid lines) and P10 (dashed lines) is indicated. Panels B, C, E, F, H, I, K, and L show the kinetics after cross-passaging. The permissivity (for control cells lacking cross-propagation) is presented in panel M, where the *y*-axis (labeled as log TCID_50_ per mL increase) represents the permissivity of the cell line for CCHFV. An increase in permissivity is characterized by a curve with a positive slope. Error bars represent standard deviation (SD).

Regarding infectious titers, serial passaging did not result in increased maximum TCID_50_ values (Fig. 2, Supplementary Table S3). The highest infectious titer was observed in Vero cells during P10. Notably, the highest variation in the infectious titers occurred when the virus was cross-passaged from Vero E6 cells to other cell lines.

We also performed quantification of the M and L segments from the supernatant of passages P1 and P10 (at 0 and 7 dpi) and compared the increase in the log titer of viral RNA between the cell lines and passages (Supplementary Fig. S3). The highest increase was seen in the M segment (2.05 ± 0.678 log in average) in all cell Lines. The log titer increase between 0 and 7 dpi showed moderate cell line dependence (*P* = 0.0628) and was also affected by passage number (*P* = 0.036). The log titer increase of the L segment vRNA also showed cell line dependence (*P* = 0.0008) and was influenced by serial passaging (*P* = 0.0066).

Based on the viral growth kinetics observed during P1, P6, and P10, we determined the day postinfection at which the highest TCID_50_ per mL was observed for each sample (Figs. 1 and 2A-L, Supplementary Table S3). The maximum infectious titers were reached 24–48 hours earlier during P10 when compared to P1 and P6. Consequently, we calculated the logarithmic increase in the infectious titers at an MOI of 0.005 for the day postinfection with the highest TCID_50_ per mL during P10 (Fig. 2, dashed lines; the respective days postinfection are listed in Supplementary Table S3). Permissivity was assessed based on the TCID_50_ per mL at the selected time point (Vero E6, day 4; Vero, day 5; SW13, day 2; BHK-21, day 2) when the virus was serially passaged in the same cell line. Our results indicated that the highest initial permissivity (at P1) was found in SW13 cells (a 3.83-log increase in the TCID_50_ by day 2). However, this did not increase by P10. The most significant increase in permissivity (2.5 log on average) between P1 and P10 was observed when the virus was passaged in Vero cells, suggesting adaptation to Vero cells following cross-passaging from other cell lines. For Vero E6 cells, the initial permissivity was higher compared to Vero cells (a 2.99-log increase in the TCID_50_ by day 4), and serial passaging resulted in a further increase in permissivity over time (an average increase of 1.0 log). The lowest initial permissivity was observed in BHK-21 cells (a 1.25-log increase in TCID_50_ by day 2), with an average increase of 2.25 log following serial passaging.

We also assessed the particle (viral RNA copy number)-to-TCID_50_ per mL ratio during P1 and P10 (Supplementary Fig. S2). In SW13 cells, the ratio (6.76 × 10^3^; determined on day 2 postinfection) did not decrease with serial passaging, indicating a constant ratio of infectious particles within the viral cloud. In Vero E6, Vero, and BHK-21 cells, the ratio (initial ratios, 6.17 × 10^3^, 1.72 × 10^3^, and 1.21 × 10^5^, respectively) decreased by an average of 0.47, 0.69, and 0.99 log, respectively, indicating that the relative ratio of infectious particles increased after 10 rounds of passage. Interestingly, in Vero E6 cells, the ratio reached its maximum one day later (4 dpi) during P10, compared to P1 (3 dpi). In contrast, in BHK-21 cells, the ratio peaked 24 hours earlier (2 dpi) after 10 passages, compared to P1 (3 dpi).

### Distribution of acquired mutations in the viral genome

The overall average genome coverage with sequencing depth over 100x was 99.55% (91.95–100%) in the coding regions. During the study, we identified a total of 953 SNPs, including 256 unique mutations with a frequency over 10%. The average variant frequency (VF) based on all samples was 16.35% (95% CI 11.66–21.04%), 15.53% (95% CI 12.87–18.18%), and 33.74% (95% CI 27.43–40.04%) in the S, M, and L segment, respectively.

**Fig. 3:**
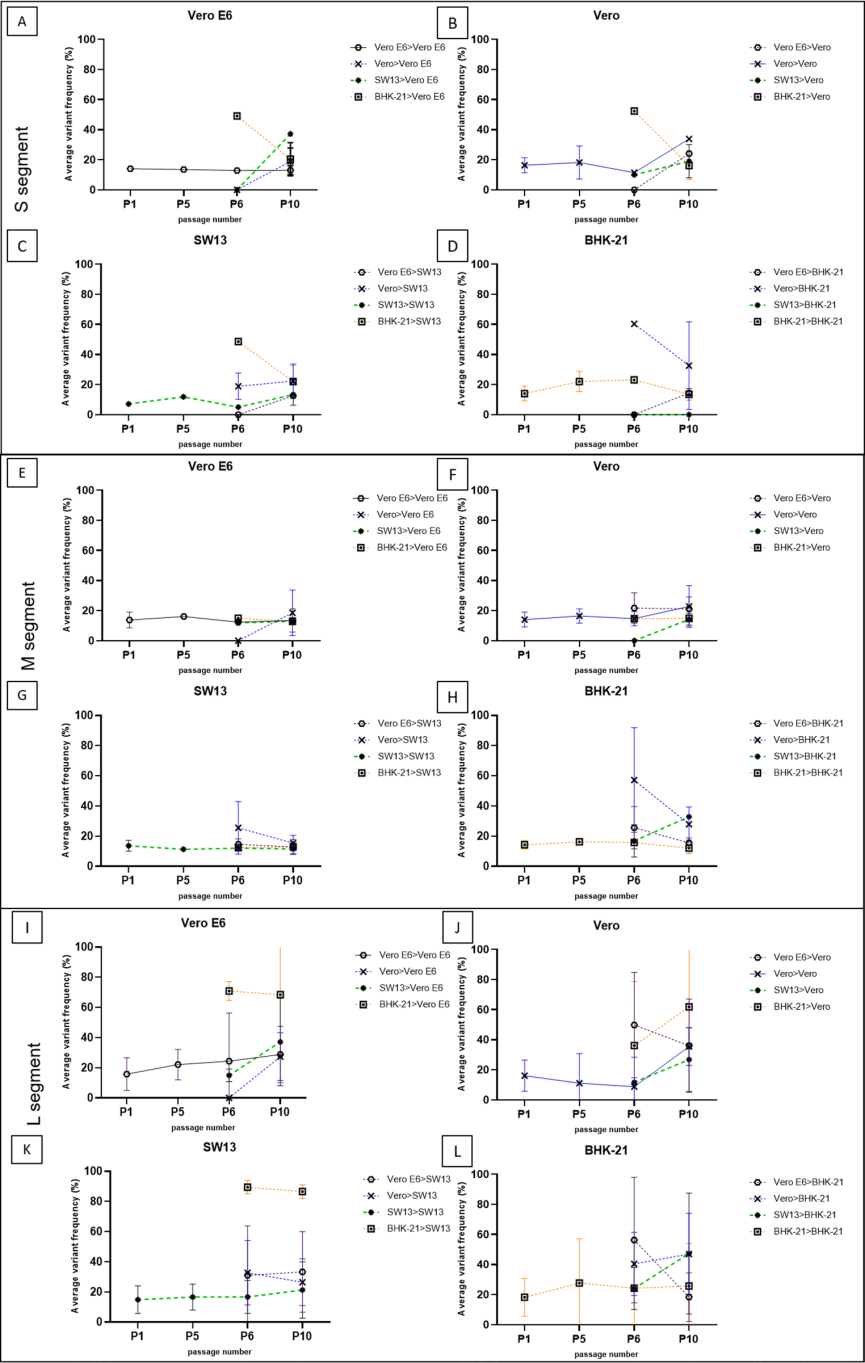
Distribution of all identified variations with a frequency over 10% along the whole genome of CCHFV for the P1, P5, P6, and P10 samples. (**A**) S segment. (**B**) M segment. (**C**) L segment. Variant frequencies were determined based on the sequencing data of parallel biological replicates pooled together prior to library preparation. The first column shows the P1 (green) and P5 (orange) passage results by cell Line. Columns 2–5 represent P6 (blue) and P10 (purple) cross-passages, indicated as original cell Line > new cell Line. Mutations of particular interest emerging with a frequency over 40% (indicated by a dashed line) are highlighted by red arrows.

In the S segment, the average VF increased from 11.12% (P1) to 19.61% (P10) (Fig. 3A-D). In the M segment, the average VF was 13.93% (P1) and increased to 16.27% (in the range of 13.20% in SW13 and 19.17% in BHK-21, Fig. 3E-H) by P10, significantly depending on the new cell line (*P* = 0.0301). In the L segment, the average VF showed a strong increase from 16.27% (P1) to 39.23% (P10) (*P* < 0.0001) during serial passaging (Fig. 3I-L). The number of SNPs with a frequency over 10% was 1.2, 1.77, and 1.13 per kb in the S, M, and L segment, respectively.

**Figure 3** Average variant frequency by genome segment and cell line used for propagation. New cell lines after cross-passaging are shown in separate panels (A-L), and the previous (original) cell lines are represented by colors. Data are shown for each passage (P1, P5, P6, and P10). Error bars represent standard deviation (SD).

We also determined the distribution of all identified nucleotide variations over 10% frequency along the genome segments by genome position for each sample (Fig. 3). Variants within the P0 inoculum of the CCHFV stock were also examined in order to set the genetic baseline and track changes in the subclonal diversity of the virus, but these were excluded from the visualization.

In the S segment (Fig. 3A), we found two individual nt variations with frequencies over 40% (highlighted by red arrows). Both variations emerged when the virus was cross-passaged from BHK-21 cells or from Vero to BHK-21 cells. Although the average VF did not show strong cell specificity in this segment (*P* = 0.167, Fig. 3A-D), the number of mutations increased (with 0-2.5 nt variations per kb, depending on the cell line) during serial passaging, especially when passaged in Vero E6 or SW13 cells (Fig. 3 A and C, Fig. 3A).

In the M segment (Fig. 3E-H), a constant average VF was found during serial passaging that was independent of the passage number (*P* = 0.0556), but it was affected by the new cell line (*P* = 0.0301). Interestingly, in all of the cell lines, the number of individual SNPs was higher during P1 than in subsequent passages (Fig. 3B, first column, green dots). During P10, an increase in the number of nt variations was also observed in comparison to previous passages (Fig. 3B, purple dots). Interestingly, most of the SNPs with a frequency over 40% emerged when the virus had been passaged previously in Vero cells (Fig. 3B row 3; high-frequency SNPs are highlighted by red arrows).

In the L segment (Fig. 3I-L), the VF increased significantly by P10 when compared to P1 (16.26–27.02% increase on average, *P* < 0.0001), in addition to the large number of SNPs seen during the first passage (Fig. 3C, green dots, 35–41 nt variations, depending on the cell line), which decreased during the subsequent passages. This indicates the rapid emergence of low-frequency quasispecies during P1 and positive selection and fixation of certain consensus-level mutations in the L segment due to serial passaging and possible adaptation to the cell lines.

### Analysis of nucleotide change preferences

Nucleotide changes that occurred in over 10% of the variants were identified in all three genome segments (Supplementary Fig. S4). In the inoculum stock, which had previously been amplified in Vero E6 cells, 18 high-frequency SNPs were identified in comparison to the reference sequence of the CCHFV Afg09-2990 strain, including four (G > A, T > C, and C > T) variations in the S segment, four (A > G, G > A, and T > A) in the M segment, and 10 (A > G, A > T, C > T, T > A, and T > C) in the L segment.

After further passaging, the most common high-frequency nucleotide change in the S segment was T > C for all cell lines (42.11%, 95% CI: 32.64–51.57%). A > C nt changes occurred specifically when the virus was passaged in Vero E6 cells (18.42%, 95% CI 8.96–27.88%), and T > G nt changes occurred most frequently when the virus was passaged in BHK-21 cells (15.79%, 95% CI 6.33–25.25%).

In the M segment, the most common nucleotide changes were A > C and A > G, with a frequency of 24.76% (95% CI: 20.21–29.32%) and 20.95% (95% CI: 16.40-25.51%), respectively, in all of the cell lines. Interestingly, C > A changes were more abundant in the viral genome when the virus was passaged in Vero E6 or SW13 cells.

No high-abundance cell-line-specific nucleotide change preferences were seen in the L segment; the most common SNPs were T > C, with a frequency of 26.33% (95% CI: 21.69–30.96%) and A > G, with a frequency of 23.18% (95% CI: 18.55–27.82%). Interestingly, C > A nt changes occurred only when the virus was passaged in SW13 cells (1.38%, 95% CI 0-6.01%), whereas C > G (3.73%) and T > G (8.25%) nt variations occurred most frequently in BHK-21 cells.

### Annotation of consensus-level nucleotide variations

Nucleotide variants with a frequency above 40% were annotated and compared to the consensus sequence of the P0 inoculum virus stock (Table 1). We found two SNPs in the S segment, one of which was a probable consequence of adaptation to the BHK-21 cell line (see also Fig. 3A). The silent mutation I448I (nt 1399, ATT > ACC) was present in the P0 virus genome with a frequency of 3.65%, but when the virus was passaged in BHK-21 cells, the frequency increased to 26.51% by P5. After cross-passaging in Vero E6, Vero, and SW13 cells, the average frequency was 42.4–52.4% during P6, depending on the cell line, and the mutation persisted through P10. The VF was between 26.9 and 37.2%, depending on the cell line (Fig. 3A, row 1).

**Table 1:** Annotation of the nucleotide variants with frequency over 40%. aa, amino acid; nt, nucleotide

aa change	nt position	Average depth of coverage	nt change	Type	Genome region	Relevance
S segment						
I448I	1399	2855	ATT > ACT	Silent	NP head domain	Quasispecies emerged in BHK-21 cells; mutation fixed in the viral genome and persists when passaged in other cell lines.
M segment						
L276L	920	12041	TTA > TTG	Silent	GP38	Quasispecies emerged in Vero cells; mutation fixed in the viral genome and persists when passaged in other cell lines. Slow reversion in SW13 cells
E594K	1872	9260	GAG > AAG	Non-silent	Gn	Quasispecies emerged in Vero cells, and adapted to Vero E6, Vero and BHK-21 cells. Slow reversion in SW13 cells
D1168G	3595	2515	GAC > GGC	Non-silent	Gc	Low-frequency quasispecies emerged in Vero E6 and persisted in Vero E6, BHK-21, and SW13 cells with low frequency, and in Vero cells with high frequency.
L segment						
D618E	1931	3282	GTA > GCA	Non-silent	Zinc-finger (ZF) domain	Potential NP-binding function alteration; BHK-21-specific virus adaptation, persisting as quasispecies in the other cell lines
R837S	2588	10902	AGA > AGT	Non-silent	NP-binding site	Vero-E6- and SW13-specific virus adaptation
P889P	2774	6422	CCT > CCC	Silent	NP-binding site	BHK-21-specific virus adaptation, persisting as quasispecies in the other cell lines
E1093E	3352	7137	GAA > GAG	Silent		Present in the P0 genome; emerged in Vero cells specifically, reverted in BHK-21 cells
A1159T	3552	6839	GCA > ACA	Non-silent		Vero-cell-specific persisting quasispecies, but reverts in other cell lines
N2025N	6152	3103	GAC > GAT	Silent		Vero-E6- and SW13-specific virus adaptation
A2279V	6913	5191	GCA > GTA	Non-silent	RdRp motif	Potential catalytic activity alteration; present in the P0 genome; emerged in all cell lines as quasispecies but strong adaptation to BHK-21. Reverts in SW13 cells
F2576F	7805	8832	TTT > TTC	Silent	RdRp motif	BHK-21-specific nt variation
T3667A	11076	52363	ACC > GCC	Non-silent	NP-binding site	Vero-specific nt variation
A3879V	11713	63847	GCA > GTA	Non-silent	NP-binding site	Vero-E6- and SW13-specific nt variation

In the M segment, we found no variations with a frequency higher than 40% during the first five serial passages (Fig. 3B, column 1). After cross-passaging, likely adaptation to a specific cell line was observed in the case of three mutations (out of six unique SNPs). The silent mutation L276L (nt 920, TTA > TTG) in the GP38 region of the M segment emerged in Vero cells, reaching a frequency of 24.3% by P5, and persisted further in the Vero (50.3%), Vero E6 (53.47%), and BHK-21 (99.5%) cells until P10. The mutation showed slow reversion in SW13 cells, decreasing to a frequency of 12.4% by P10. The mutation E594K (nt 1872, GAG > AAG) in the Gn coding region emerged when the virus was passaged in Vero cells (P5: 23.80%), and it emerged in Vero E6 (46.0%), Vero (44.5%), and BHK-21 (64.7%) cells but reverted in SW13 cells (33.3% by P6 and 10.6% by P10). The mutation D1168G (nt 3595, GAC > GGC) in the Gc region emerged when the virus was passaged in Vero E6 cells and persisted with a frequency around 10% when serially passaged in Vero E6, SW13, or BHK-21 cells. The mutation was present at a higher frequency (54.5%) by P10 when passaged in Vero cells. Other high-frequency mutations identified in the M segment showed no cell-line specificity, but there were mainly A > G, G > A, T > A, and C > T nucleotide changes.

In the L segment, a total of 16 individual SNPs with a frequency of more than 40% were observed, 10 of which were associated with a specific cell line. Two nt variations were present in the original P0 sequence as ambiguities. One of these, A2279V (nt 6913, GTA > GCA) in the RdRp region, occurred with a frequency of 77.11% and further persisted in all cell Lines with a frequency of 12.06–100.0%, depending on the cell line. A2279V emerged with high VF when passaged in BHK-21 cells (over 90.0%) but showed reversion after further passaging in SW13 cells. The silent mutation E1093E (nt 3352, GAG > GAA), with a 42.17% frequency in the P0 genome, persisted further with high frequency (over 53.0%) when the virus was passaged in Vero cells. E1093E showed complete reversion in BHK-21 cells. The mutation D618E (nt 1931, GAT > GAA) in the zinc-finger domain also emerged in all cell lines after P1 and persisted throughout long-term passaging, but with higher frequency when passaged in BHK-21 cells (over 90% frequency). Similar to the D618E mutation, P899P (nt 2774, CCT > CCC) in the NP-binding region and F2576F (nt 7805, TTT > TTC) in the RdRp motif emerged with high frequency when the virus was passaged in BHK-21 cells and persisted with further passaging in all cell lines. In addition to E1093E, two further SNPs were identified that emerged exclusively when the virus was passaged in Vero cells (A1159T and T3667A) and persisted after cross-passaging in other cell lines. In the L segment, three additional SNPs emerged that were specific to the Vero E6 and SW13 cell lines. The silent mutation N2025N (nt 6152, GAC > GAT) persisted only in Vero E6 and SW13 cells, with a frequency of 38.20–68.60%. The mutations A3879V (nt 11713, GCA > GTA) and R837S (nt 2588, AGA > AGT) both emerged in the NP-binding site as quasispecies in Vero E6 and SW13 cells and persisted until P10 in all cell Lines with over 40% VF.

## Discussion

In this study, we investigated the links between the infectivity and phenotypic properties of CCHFV and long-term genetic conservation. Because the CCHFV genome has a high mutation rate, it is particularly important to identify potential genotypic and phenotypic changes that occur during routine propagation of this virus *in vitro* [7, 8].

We designed an experiment to examine the mutations that accumulated during serial passage of the virus over 10 generations, using whole-genome sequencing to map mutations and identify variants that were selected in different cell lines. It has been shown previously that CCHFV can infect a wide range of cell lines, but the changes that occur due to serial passaging and adaption have not been studied before [13, 14, 18–22]. Here, we investigated the effect of using three different MOI values during the first infection (0.005, 0.01, 0.1) in four different cell lines that are commonly used for CCHFV propagation (Vero E6, Vero, SW13, and BHK-21 cells, Fig. 1).

Our results showed that the maximum virus copy number per mL was independent of the initial MOI, as it increased to the same level due to the limiting effect of the number of susceptible cells. Furthermore, defective interfering particles (DIPs), which are products of spontaneous error-prone virus replication of RNA viruses, are known to have an inhibitory effect on viral growth. DIPs play an important role during *in vitro* and *in vivo* infection, interfering with active virus replication and promoting persistence, especially when infection is initiated at a high MOI. Therefore, the emergence of DIPs during replication can influence the logarithmic increase in virus titers [23]. Consistent with this, we found that the logarithmic increase in the viral RNA copy number was the highest when the lowest MOI (0.005) was used for infection of the cells. We also compared the maximum infectious titer for each cell line and found that the highest titer was obtained with SW13 cells. Furthermore, we detected CPE only in SW13 cells, which was consistent with the findings of Dai et al., Li at al., and Agol et al. [13, 14, 24]. Although infection of SW13 cells may induce a host-encoded necrotic program as an anti-CCHFV response, the CPE might also be attributed to host defense mechanisms that are not directly caused by viral reproduction [13]. The titer of infectious virus decreased rapidly after the onset of CPE, which is consistent with previous observations that free virus particles in culture medium are unstable at 37 °C and lose their infectivity completely within 7 hours [14, 25, 26].

Considering that the highest logarithmic increase in the infectious titers and viral RNA copies were found when cells were infected at an MOI of 0.005, we performed the subsequent serial passages (P2-P10) at this MOI, as a higher virus yield favors genetic diversity and accumulation of mutations [7]. The use of a low MOI also minimized the potential inhibitory effect of interferon carryover from previous passages, as suggested previously by Li et al. [14]. Dai et al. found that SW13, Vero, and Vero E6 cells are highly permissive, and BHK-21 is permissive for CCHFV strain YL16070 [13]. They found that the viral load (expressed as viral RNA copies per mL) decreased by 2 log after two rounds of infection in SW13 and BHK-21 cells [13]. We also found a slight decrease in viral RNA copy numbers during serial passaging (Supplementary Table S3). The initial permissivity in BHK-21 cells during P1 at an MOI of 0.005 (1.25 log TCID_50_ increase by dpi 2) was 1.42–2.58 log lower than in the other cell lines, which is consistent with the permissivity data published by Dai et al. [13]. However, it increased by an average of 2.25 log by P10 (Fig. 2M). The most significant increase in permissivity was observed in Vero cells at 5 dpi, with an average increase of 2.5 log, which may be attributed to long-term growth adaptation. Interestingly, permissivity decreased slightly in SW13 cells (Fig. 2M), as CCHFV infection at early stage in cell culture is more sensitive to interferon responses, with sensitivity decreasing once the virus begins to replicate. Because SW13 was the only cell line that exhibited CPE, enhanced interferon carryover from previous passaged might have had an impact on cell permissivity [14]. The ratio of viral-RNA-containing particles to TCID_50_ per mL during P1 and P10 was also calculated in order to assess the role of non-infectious particles that emerged during serial passaging. In SW13 cells, the ratio did not decrease with serial passaging. In Vero E6, Vero, and BHK-21 cells, the ratio decreased by an average of 0.3, 0.77, and 0.99 log, respectively, suggesting a relative increase in the number of infectious particles within the viral cloud resulting from serial passaging. The significant role of DIPs in various cell lines persistently infected with different viruses, such as respiratory syncytial virus (RSV), Ebola virus, vesicular stomatitis virus (VSV), rabies virus, and lymphocytic choriomeningitis virus (LCMV), has been highlighted in numerous publications. This has led to the assumption that a high abundance of DIPs may contribute to viral persistence by activating antiviral signaling pathways and promoting pro-survival effects in infected cells [23, 27]. DIPs may induce changes in the environment that ultimately influence the genetic diversity and evolution of the viral population as a whole. Early studies demonstrated that the genomes of VSV DIPs, as well as the parental VSV passaged for five years in persistently infected cells, undergo continuous and significant alterations throughout the passage process [28]. A more recent study further emphasized the role of DIPs in shaping viral population diversity by showing that influenza virus DIPs reduce reassortment efficiency by interfering with the generation of the wild-type virus [29]. Given that genome reassortment is a major driver of genetic diversity in segmented viruses such as influenza virus and CCHFV, DIPs appear to play a critical role in suppressing viral diversification through genetic shift.

In order to map genetic variations that occurred during cell culture passage, whole-genome sequencing was performed using an unbiased SISPA enrichment method, followed by paired-end Illumina sequencing. While it has been suggested that the SISPA technique has the potential to introduce sequencing errors [17, 30, 31], it has been shown that this methodology can affect the sequencing depth in genome regions of high complexity and GC content rather than affecting the amplification bias [17, 30–33].

In our whole-genome analysis, we calculated the average VF and plotted the variant distribution by genome segment in all of the samples. Whereas a higher VF together with a small number of mutations suggests host adaptation and a gain of fitness, a lower VF together with a large number of mutations could indicate the persistence of quasispecies serving as the basis for the selection of virulent variants [34]. The highest increase in the average VF over the course of serial propagation was seen when the virus was passaged in BHK-21 cells (4.9%, Fig. 3D, H, and L), which also might explain the significant increase in the permissivity of the cell line to CCHFV (1.3 log, Fig. 2D) through host adaptation. In contrast, the average VF decreased in SW13 cells (0.42%) along with permissivity to CCHFV (0.81 log) during serial propagation.

*In vitro* growth-adaptive mutations have been described previously for other RNA viruses. Gain-of-entry function mutations were identified in the glycoprotein region of the Ebola virus Makona strain when passaged in Huh-7 and Vero E6 cells. Furthermore, a tissue-culture-specific spontaneous mutation (T544I) recognized in the Ebola virus GP showed no effect on viral pathogenesis but remained fixed in the viral genome after three passages *in vivo* [35, 36]. In the case of West Nile virus, the replicative fitness increased significantly and resulted in nonsynonymous mutations in a temperature-dependent manner when the virus was passaged 12 times in *Culex tarsalis* cells at 25 °C or 30 °C [37]. Furthermore, Nemirov et al. demonstrated for Puumala hantavirus, which also belongs to the class *Bunyaviricetes*, that mutations in the L segment and the non-coding region of the S segment emerged during serial passaging in Vero E6 cells [38]. In the present study, we also found the highest average VFs in the CCHFV L segment, with high deviation among the cell lines. Furthermore, the highest VFs were seen when the virus was passaged in BHK-21 cells. In the S segments, we found only one unique synonymous SNP (I448I) with a frequency over 40% attributed to virus propagation in BHK-21 cells. However, the overall average frequency was low (16.35%) in this segment. Nevertheless, the number of nt variations in the coding region of the S segment increased by 0-2.5 during serial passaging, indicating that variations in the viral nucleoprotein emerge at the low-frequency quasispecies level and not at the consensus sequence level (Fig. 3A). In the M segment, we found three nt variations with frequencies over 40% (L276L, E594K, and D1168G) that emerged only after changing cell lines. The average VF was the lowest in the M segment (15.53%), but the number of nt variations per kb was the highest (1.77 SNP/kb) in this segment, suggesting that propagation in different cell lines supports the emergence of low-frequency viral quasispecies in the coding region of receptor binding proteins. The overall average frequency was the highest (33.74%) in the L segment, with a low rate of mutations per kb (1.13 SNP/kb, Fig. 3I-L and Fig. 3C). The highest increase in the VF was observed when the virus was cross-passaged from BHK-21 cells, suggesting the emergence of new, high-frequency nt variants.

The biological relevance of the identified genomic variations and their role in phenotypic changes remaining to be determined, but they might enhance the permissivity of BHK-21 and Vero cells to CCHFV and decrease the viral-RNA-containing particle-to-TID_50_ ratio. Consensus-level mutations emerged in the S (I448I) and L (D618E, P889P, A2279V, F2576F) segments when the virus was propagated in BHK-21 cells, potentially contributing to increased permissivity. The nonsynonymous mutation A2279V close to the catalytic region of RdRp is particularly interesting, and its biological relevance needs further study [9]. In the M segment, all consensus-level mutations emerged when the virus was propagated in Vero cells (L276L, E594K, D1168G), which might also support enhanced permissivity. The nonsynonymous mutations E594K and D1168G emerged in the Gn and Gc region, respectively, and might have a potential effect on cell entry or virus neutralization efficacy. Furthermore, the mutation D1168G is within the glycoprotein nucleolin binding site. The biological relevance of these mutations needs further investigation [9]. It is also important to note that CPE due to virus propagation was only observed in SW13 cells, which had the highest initial permissivity. Moreover, all of the consensus-level mutations in the M segment that had previously emerged in Vero cells reverted in SW13 cells. In addition, we detected the same nt variations in the L segment when the virus was passaged in SW13 cells that were present after growth in Vero E6 cells – the cell line that was used to propagate the P0 stock. Hawmann et al. described an *in vivo* mouse-adapted variant of the CCHFV Hoti strain, which successfully mimics the severe clinical presentation of CCHFV infection in humans when tested in immunocompetent adult mice. Following 11 rounds of passaging, they identified multiple mutations in the viral genome. Notably, these mutations emerged early during serial passaging (after the fourth passage) and exhibited a VF exceeding 80%. The authors identified two mutations in the NP region, one in the NSs region, three in the M segment, and four in the L segment. Of these mutations, four were synonymous [39]. Upon comparing these mutations with those we observed in the genome of the CCHFV Afg09-2990 strain, we found no similarities, suggesting that host adaptation mechanisms may vary across different genetic clades.

To investigate the potential host adaptation further, we analyzed the nucleotide change preferences of CCHFV. It has been shown that the codon usage bias due to mutation pressure and natural selection is relatively weak in the case of CCHFV. However, some similarities have been found between the codon usage patterns of the virus and its natural hosts, such as humans and ticks, but the viral adaptation mechanisms were not investigated further [40–42]. Here, we identified T > C as the most common nucleotide change, accounting for 22.44% of all SNPs, followed by A > G, with 21.44%; both mutation types are transitions. A > C transversions were also common (13.44% overall prevalence), especially in the M segment (24.76%). The observation of growth-adaptive high-frequency nt changes when changing to a new host cell line is in agreement with the results of Rahman et al., who found the nucleotide composition of the virus to be host-dependent, based on the analysis of 179 whole-genome sequences of CCHFV derived from infected human (*Homo sapiens*), Hyalomma tick, cattle (*Bos taurus*), and sheep (*Ovis aries*) samples [40].

Silent mutations in the third nucleotide position are often acquired through adaptation mechanisms to evade the immunological response of the host organism [43]. We found that the vast majority of the silent mutations with a frequency above 40% were associated with adaptation to BHK-21 or Vero cells. One silent mutation (I448I) in the S segment emerged when passaged in BHK-21 cells but reverted in all other cell lines. In the M segment, one silent consensus-level SNP (L276L) was identified and linked to propagation in Vero cells. Two additional non-silent mutations in the Gn and Gc regions (E594K, D1168G) were specific to virus grown in Vero E6 cells. In the L segment, silent (*n* = 4) and non-silent (*n* = 6) mutations evolved in a cell-line-specific manner. Among the silent nt mutations, two emerged when passaged in BHK-21 cells (P889P, F2576F).

It is important to note some potential limitations of our study. First, the P0 inoculum stock was prepared from infected Vero E6 cells, and not from a clinical specimen. Thus, mutations associated with adaptation to Vero E6 might have appeared during previous passages. A potential countermeasure to minimize undesirable mutations and ensure the reproducibility of assays and techniques during virus propagation is to use well-characterized virus stocks, aliquoted separately, and to perform a large number of technical replicates in characterization studies and efficacy testing of vaccines or antivirals. Additionally, it is important to try to use low-passage clinical isolates that most accurately mimic the distribution of naturally occurring virus variants in the field. Secondly, the immortalized cell Lines were also passaged during the study. In order to overcome bias introduction by the cell passaging, reagents with the same lot number were used during the course of the experiment. Furthermore, since CCHFV is a tick-borne virus, incorporating a tick cell Line into our experimental design would have been advantageous. This is due to the potential species-specific characteristics in the permissiveness of CCHFV infection at the cellular level, which may differ from those observed in mammalian cell lines. Third, SISPA methodology could also affect the sequencing results. However, it has been shown that this technique might have reduced performance in regions of high complexity and GC content, rather than causing amplification bias. Finally, we limited our analysis to nt variants that were present in at least 10% of all reads in the regions with a sequencing depth of at least 100x, resulting in some data loss. Furthermore, variant frequencies were determined based on the sequencing data of parallel biological replicates pooled together prior to barcoding, which precluded the analysis of mutation patterns across individual replicates.

In conclusion, we studied phenotypic and genotypic changes in the CCHFV genome in a stable and controlled cellular environment. The combined use of serial passaging and next-generation sequencing allowed us to identify mutations at low frequency arising across the entire viral genome, as expected for RNA viruses with a high error rate during replication. By focusing on the mutations that reached consensus-level frequency concomitant with the adapted phenotype, we were able to combine viral evolution in a controlled laboratory setting with phenotypic characterization in order to better understand *in vitro* propagation of CCHFV and its possible adaptation to commonly used cell lines, which might affect future approaches to development of novel diagnostic tests, antiviral drugs, and vaccines.

## Electronic Supplementary Material

Below is the link to the electronic supplementary material

Supplementary Material 1 (DOCX 1.10 MB)

## Data Availability

Quality-trimmed sequencing reads generated in this study are available in the Sequence Read Archive (SRA) database under the BioProject ID: PRJNA1198197 at the following link: http://www.ncbi.nlm.nih.gov/bioproject/1198197.
