# Supplementary material for: Phenotypic and genetic changes of Crimean-Congo hemorrhagic fever virus during serial passage in susceptible cell lines
Source: Arch Virol. 2025 Nov 7;170(12):240. doi: 10.1007/s00705-025-06420-4 (PMC12594681; doi:10.1007/s00705-025-06420-4)
Supplement: Supplementary file 1 — Supplementary Material 1 (DOCX 1.10 MB) [file 705_2025_6420_MOESM1_ESM.docx]

**Supplementary material**

**Investigating the growth-induced phenotypic and genetic changes of Crimean-Congo hemorrhagic fever virus in susceptible cell lines**

Nóra Deézsi-Magyar^1,2^, Bereniké Novák^1^, Gyula Zsidei^1^, Norbert Solymosi^3^, Marianna Mezősi-Csaplár^1^, Dániel Déri^1^, Bernadett Pályi^1+^ & Zoltán Kis^1,4+*^

^1^ National Biosafety Laboratory, National Center for Public Health and Pharmacy, Budapest, Hungary

^2^ School of PhD Studies, Semmelweis University, Budapest, Hungary

^3^ University of Veterinary Medicine Budapest, Budapest, Hungary

^4^ Institute of Medical Microbiology, Faculty of Medicine, Semmelweis University, Budapest, Hungary

^+^ These authors contributed equally to this work.

^*^ Correspondence: Dr. Zoltán Kis, e-mail: kis.zoltan@semmelweis.hu, kis.zoltan@nngyk.gov.hu

**
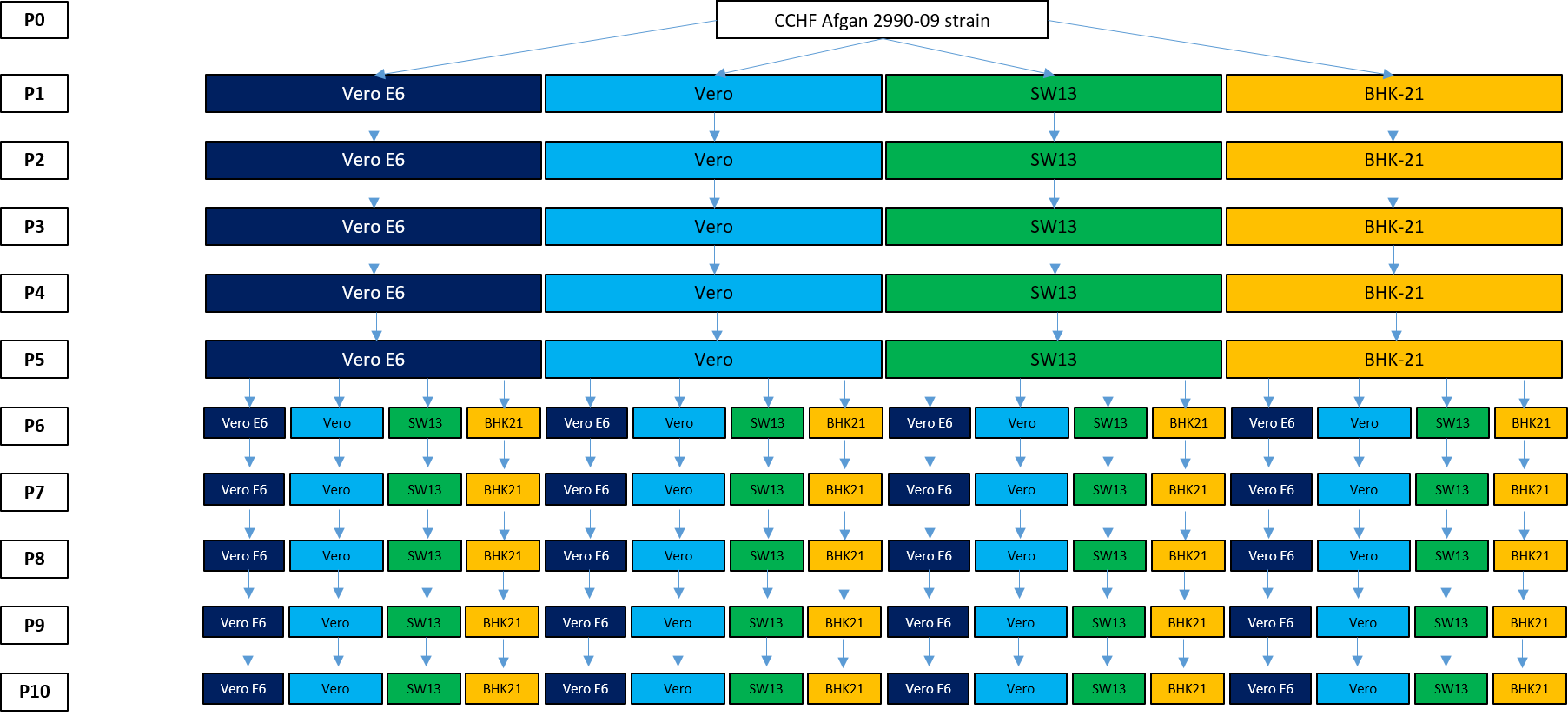
**

**Supplementary Figure 1. Our mutation accumulation study design.** For the first passage (P1), suspensions of all four cell lines (at an initial 3,0E+05 cells/well density) were inoculated in three biological and three technical replicates by using three different multiplicity of infection values (MOI 0.005, 0.01 and 0.1). Supernatant of each sample was collected directly after inoculation (day post infection; dpi 0), then in every 24 hours until day 7 (dpi 0-7). Based on virus growth, the optimal MOI (0.005) and dpi for harvesting the supernatant were determined and used for the subsequent passages. After the first passage, the virus was further propagated in the same cell lines for an additional four passages (P1 - P5). Thereafter, the virus was further cross-passaged in every cell line for five additional times (P6 - P10). During the first cross-passage (P6) and the final passage (P10) the viral growth kinetics was also determined as described above. CCHFV whole genome sequencing was performed from the samples of P0, P1, P5, P6 and P10.

**
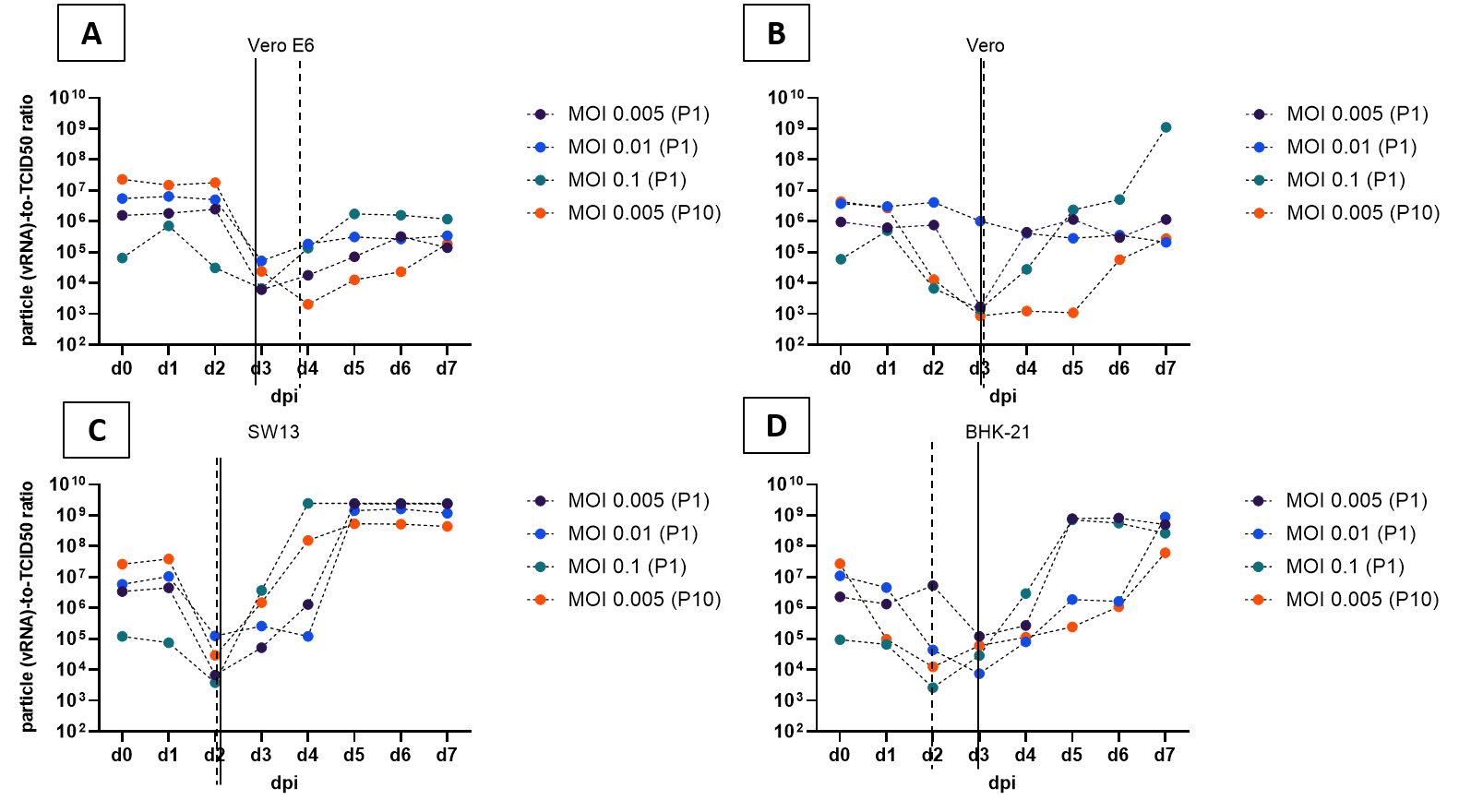
**

**Supplementary Figure 2. Calculated particle (virus RNA copy number)-to-TCID50 per mL ratio during P10 at all three MOIs, and P10 (MOI 0.005).** The ratio was calculated on each dpi. Low SD among replicates is not visible on the bar despite the visualization. Highest ratios at MOI 0.005 during P1 (solid lines) and P10 (dashed lines) are indicated.


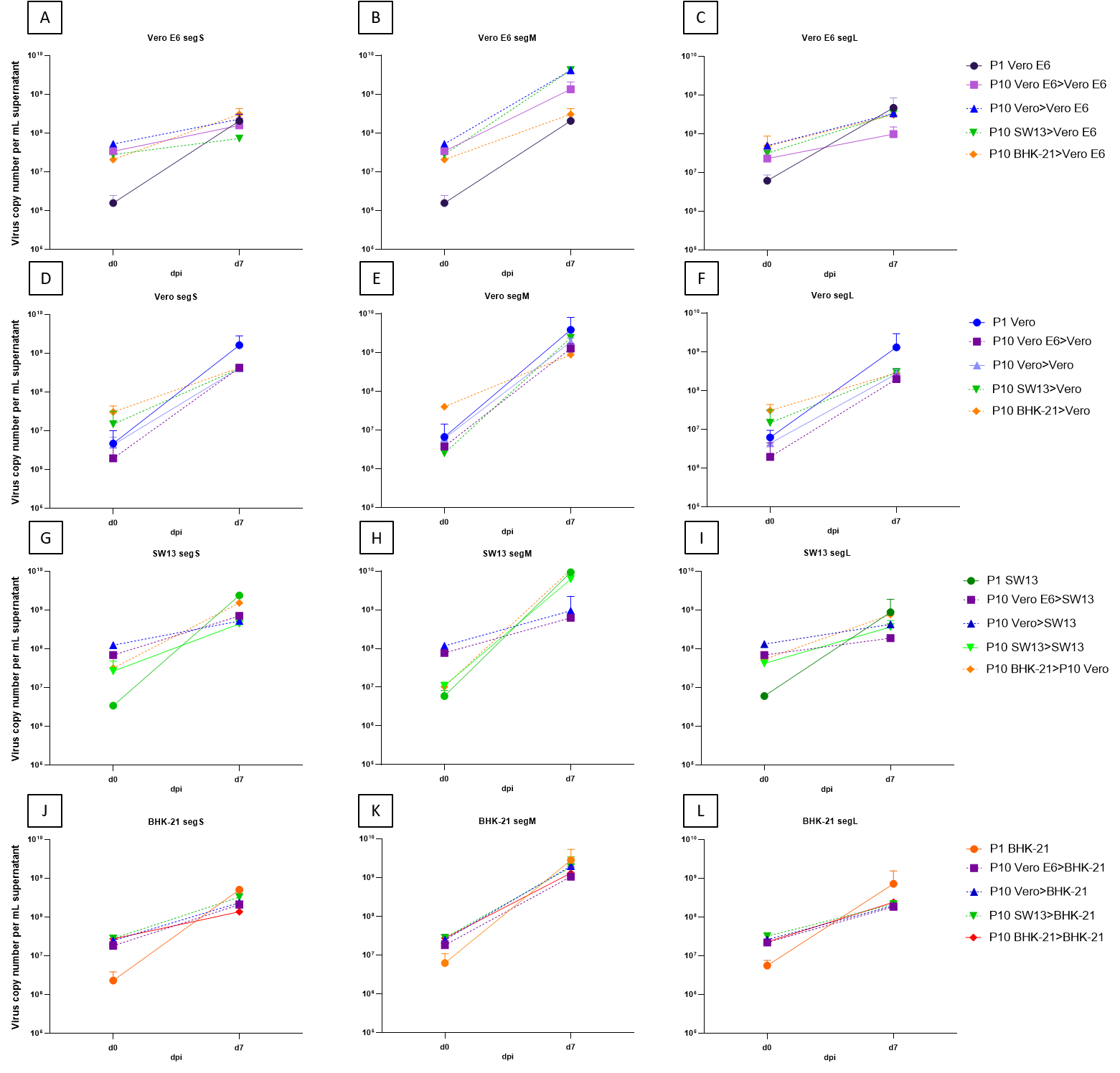


**Supplementary Figure 3. CCHFV vRNA copy number per mL supernatant for quantifying the S, M and L segments.** vRNA copy number per mL supernatant was determined at inoculation (dpi 0) and at the final kinetic time point (dpi 7) during P1 and P10. Error bars represent standard deviation (SD) among biological replicates. Cell lines are indicated in light (P1) and dark (P10) colors (Vero E6: purple, Vero: blue, SW13: green, BHK-21: orange).


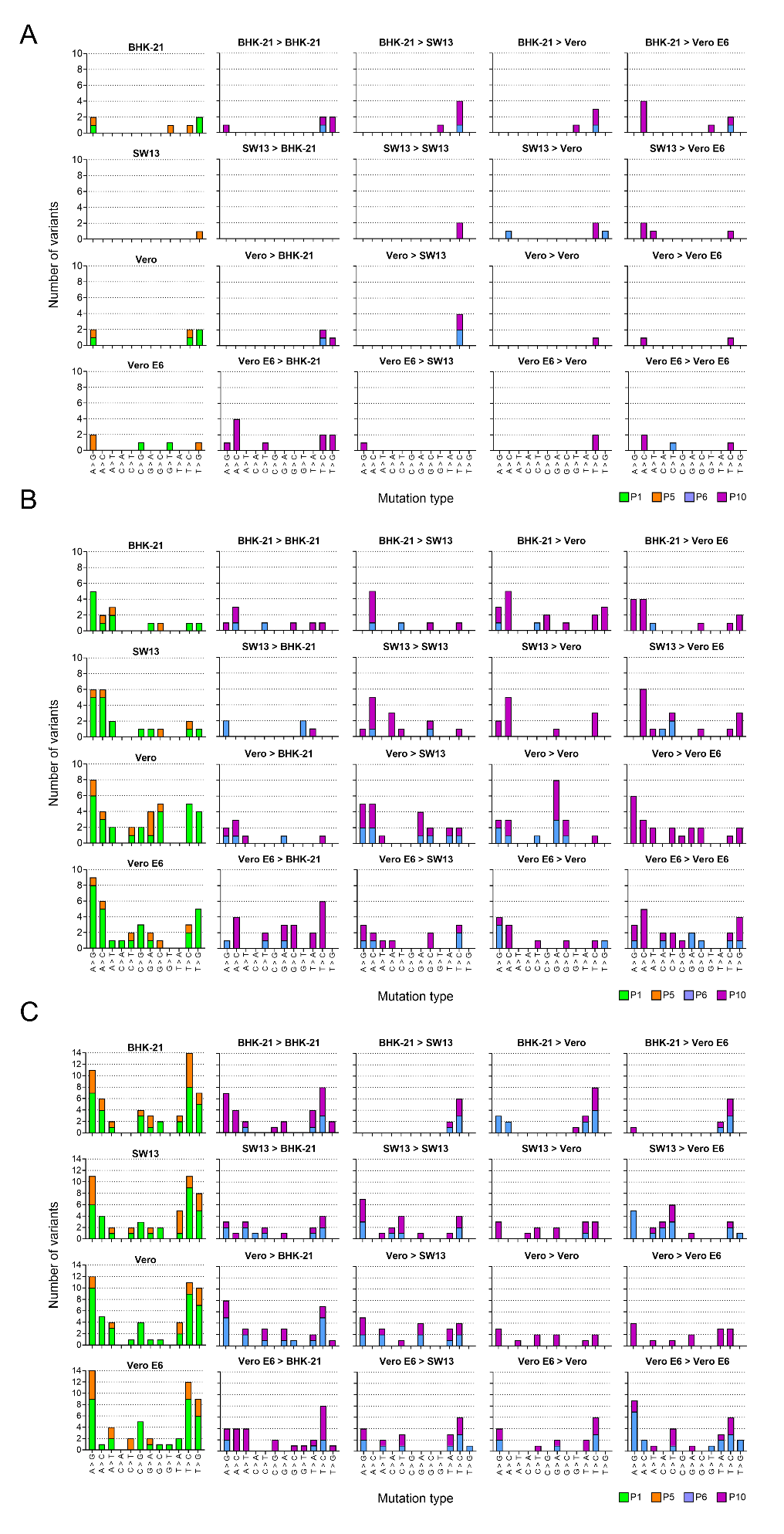


**Supplementary Figure 4. Nucleotide change preferences in the CCHFV genome induced by the host cell line by each segment: S (A), M (B) and L (C).** Nucleotide change types are shown on the x-axis. First columns show the P1 (green) and P5 (orange) passage results by cell line. Columns 2-5 represent P6 (blue) and P10 (purple) cross-passages indicated as original cell line>new cell line: columns 2. Passaged to BHK-21, column 3. to SW13, column 4. to Vero, column 4. Vero E6 cells.

**Supplementary Table 1.** Primers and probes targeting the M and L segments of CCHFV.

| **Name** | **Sequence (5'-3')** |
| --- | --- |
| M_421F | CCGATTCTAGCACACAGGCA |
| M_485P | CAGCCCTAGCACACCATCC |
| M_530R | **FAM-**CACGGGATAGTGTGTGCCTT**-BHQ** |
| L_7824F | GGCTACAGAGTGACTCCTGC |
| L_7879P | GCTCTGTAACCTCTCCTCAGAG |
| L_7933R | **FAM-**TGTTGGGATGAAACCTGGCA-**BHQ** |

**Supplementary Table 2. Virus growth descriptive measures obtained during P1 in the different cell lines** (maximum virus RNA copy number per mL, infective titer described as TCID_50_ per mL, logarithmic increase in virus growth between dpi 0 and dpi with maximum virus RNA copy numbers and infective titers, and dpi detected with the highest viral load).

| **P1** | **MOI 0.005** | | | **MOI 0.01** | | | **MOI 0.1** | | |
| --- | --- | --- | --- | --- | --- | --- | --- | --- | --- |
| **Virus RNA copy number per mL** | **maximum** | **Logarithmic increase** | **dpi** | **maximum** | **Logarithmic increase** | **dpi** | **maximum** | **Logarithmic increase** | **dpi** |
| **Vero E6** | 4.88E+08 | 2.49 | 5 | 5.03E+08 | 1.96 | 5 | 5.57E+08 | 1.43 | 5 |
| **Vero** | 1.25E+09 | 2.84 | 6 | 1.44E+09 | 2.48 | 6 | 1.62E+09 | 1.93 | 6 |
| **SW13** | 2.39E+09 | 2.84 | 5 | 1.62E+09 | 2.44 | 5 | 2.44E+09 | 1.80 | 4 |
| **BHK-21** | 8.23E+08 | 2.55 | 5 | 8.92E+08 | 1.91 | 6 | 7.13E+08 | 1.37 | 6 |
| **TCID_50_ per mL** | **maximum** | **Logarithmic increase** | **dpi** | **maximum** | **Logarithmic increase** | **dpi** | **maximum** | **Logarithmic increase** | **dpi** |
| **Vero E6** | 6.81E+03 | 3.83 | 5 | 1.47E+03 | 3.17 | 6 | 3.16E+03 | 1.00 | 3 |
| **Vero** | 3.16E+03 | 3.80 | 6 | 3.16E+03 | 3.50 | 7 | 3.16E+04 | 2.00 | 3 |
| **SW13** | 3.16E+04 | 4.50 | 3 | 6.81E+03 | 3.83 | 4 | 6.81E+04 | 2.33 | 2 |
| **BHK-21** | 4.47E+03 | 3.65 | 4 | 6.81E+03 | 3.83 | 3 | 3.16E+04 | 2.00 | 2 |

**Supplementary Table 3.** Maximum virus RNA copy number per mL and maximum infective titer (TCID_50_ per mL) by original cell lines (P1-P5) and after the cross passages (P6-P10) to the new cell lines. Dpi when infective titers were determined by original cell lines and new cell lines during P1, P6 and P10.

| **Maximum virus RNA copy number per mL** | | | | | | | | |
| --- | --- | --- | --- | --- | --- | --- | --- | --- |
| **new cells (P6-P10)** | **Vero E6** | | | | **Vero** | | | |
| **original cells (P1-P5)** | **Vero E6** | **Vero** | **SW13** | **BHK-21** | **Vero E6** | **Vero** | **SW13** | **BHK-21** |
| **P1** | 5,43E+08 |  |  |  |  | 1,25E+09 |  |  |
| **P6** | 2,77E+07 | 2,01E+08 | 2,41E+08 | 6,15E+08 | 1,31E+08 | 1,29E+08 | 2,16E+08 | 3,15E+08 |
| **P10** | 1,58E+08 | 2,74E+08 | 7,28E+07 | 3,08E+08 | 4,23E+08 | 4,09E+08 | 4,03E+08 | 4,23E+08 |
| **new cells (P6-P10)** | **SW13** | | | | **BHK-21** | | | |
| **original cells (P1-P5)** | **Vero E6** | **Vero** | **SW13** | **BHK-21** | **Vero E6** | **Vero** | **SW13** | **BHK-21** |
| **P1** |  |  | 2,39E+09 |  |  |  |  | 9,67E+08 |
| **P6** | 1,13E+09 | 1,13E+09 | 9,90E+08 | 8,65E+08 | 2,62E+08 | 2,14E+08 | 1,98E+08 | 3,55E+08 |
| **P10** | 7,01E+08 | 7,01E+08 | 5,42E+08 | 1,62E+09 | 2,10E+08 | 2,36E+08 | 3,28E+08 | 1,81E+08 |
| **Maximum TCID_50_ per mL** | | | | | | | | |
| **new cells (P6-P10)** | **Vero E6** | | | | **Vero** | | | |
| **original cells (P1-P5)** | **Vero E6** | **Vero** | **SW13** | **BHK-21** | **Vero E6** | **Vero** | **SW13** | **BHK-21** |
| **P1** | 6,81E+04 |  |  |  |  | 6,81E+03 |  |  |
| **P6** | 4,38E+02 | 4,38E+02 | 4,38E+02 | 4,38E+02 | 2,21E+03 | 2,21E+03 | 2,21E+03 | 2,21E+04 |
| **P10** | 7,01E+03 | 7,01E+03 | 1,21E+03 | 2,78E+04 | 2,72E+05 | 5,59E+04 | 2,72E+04 | 4,38E+03 |
| **new cells (P6-P10)** | **SW13** | | | | **BHK-21** | | | |
| **original cells (P1-P5)** | **Vero E6** | **Vero** | **SW13** | **BHK-21** | **Vero E6** | **Vero** | **SW13** | **BHK-21** |
| **P1** |  |  | 3,16E+04 |  |  |  |  | 3,16E+03 |
| **P6** | 1,26E+04 | 3,16E+03 | 1,26E+03 | 2,12E+04 | 3,16E+03 | 3,16E+03 | 3,16E+03 | 1,26E+04 |
| **P10** | 4,38E+02 | 1,21E+03 | 6,44E+03 | 1,39E+04 | 5,59E+03 | 3,16E+03 | 2,33E+03 | 2,60E+03 |
| **dpi used for determination of infective titers (TCID_50_ per mL)** | | | | | | | | |
| **new cells (P6-P10)** | **Vero E6** | | | | **Vero** | | | |
| **original cells (P1-P5)** | **Vero E6** | **Vero** | **SW13** | **BHK-21** | **Vero E6** | **Vero** | **SW13** | **BHK-21** |
| **P1** | 5 |  |  |  |  | 6 |  |  |
| **P6** | 6 | 5 | 5 | 5 | 6 | 6 | 7 | 7 |
| **P10** | 4 | 4 | 4 | 3 | 4 | 5 | 5 | 4 |
| **new cells (P6-P10)** | **SW13** | | | | **BHK-21** | | | |
| **original cells (P1-P5)** | **Vero E6** | **Vero** | **SW13** | **BHK-21** | **Vero E6** | **Vero** | **SW13** | **BHK-21** |
| **P1** |  |  | 3 |  |  |  |  | 4 |
| **P6** | 3 | 4 | 2 | 2 | 4 | 6 | 4 | 4 |
| **P10** | 2 | 2 | 2 | 2 | 3 | 4 | 2 | 2 |
